# Immunotherapy with cure potential of multi-drug resistant hematologic malignancies using IL-2 preactivated intentionally mismatched donor lymphocyte

**DOI:** 10.1007/s00432-023-04780-5

**Published:** 2023-05-19

**Authors:** Shimon Slavin

**Affiliations:** 1 Biotherapy International, The Center for Cancer Immunotherapy & Cellular Medicine, Weizmann Center, 14 Weizmann Street, 64239 Tel Aviv, Israel; 2 Stem Cell Transplantation & Cancer Immunotherapy Center, Hadassah Medical Center, Jerusalem, Israel

**Keywords:** Hematologic malignancies, Leukemia, Non-Hodgkin lymphoma, Multiple myeloma, Immunotherapy, Cell-mediated immunotherapy, Mismatched donor lymphocytes, IL-2 activated killer cells, Multi-drug resistant cancer

## Abstract

**Purpose:**

Unfortunately, cure of multi-drug resistant (MDR) hematologic malignancies remains an unmet need. Donor lymphocyte infusion (DLI) following allogeneic stem cell transplantation (SCT) can sometimes eliminate multi-drug resistant leukemia but at a risk of acute and chronic graft-vs-host disease (GVHD) and procedure-related toxicity. Supported by pre-clinical experiments in animal models, we hypothesized that immunotherapy induced by non-engrafting intentionally mismatched IL-2 activated killers (IMAK) including both T & NK cells could induce safer, faster and much more effective immunotherapy while avoiding the need for SCT and the risks of GVHD.

**Methods:**

IMAK treatment was applied in 33 patients with MDR hematologic malignancies conditioned with cyclophosphamide 1000 mg/m^2^ based protocol. Haploidentical or unrelated donor lymphocytes were preactivated with IL-2 6000 IU/ml for 4 days. IMAK was combined with Rituximab in 12/23 patients with CD20^+^ B cells.

**Results:**

A total of 23/33 patients with MDR (4 failing SCT) achieved complete remission (CR). First patient currently 30 years with no further treatment and 6 observed for > 5 years (2 AML; 2 multiple myeloma, 1 ALL & 1 NHL) can be considered cured. No patient developed > grade 3 toxicity or GVHD. No residual male cells were detectable among six females treated with male cells beyond day + 6, confirming that GVHD was prevented by consistent early rejection of donor lymphocytes.

**Conclusions:**

We hypothesize that safe and superior immunotherapy of MDR with cure potential may be accomplished by IMAK, most probably in patients with low tumor burden, but that remains to be confirmed by future clinical trials.

## Introduction

Although most patients with hematological malignancies respond initially to conventional anti-cancer modalities, primary resistant and recurrent disease represent the major causes of failure of conventional modalities and allogeneic stem cell transplantation (SCT). We have pioneered the use of donor lymphocytes infusion (DLI) for treatment of relapse following myeloablative SCT as early as 1986, confirming the feasibility to eliminate chemo-radiotherapy-resistant leukemia in a proportion of multi-drug resistant patients using alloreactive donor lymphocytes (Slavin and Nagler 1991; Slavin et al. 1995, 1996). The effective role of DLI for induction of graft-versus-leukemia (GvL) effects was subsequently confirmed by many transplant centers worldwide (Collins et al. 1997; Kolb et al.^1995^; Mackinnon et al. 1995; Takahiro et al. 2017). The use of graded increments of DLI following SCT while watching for early signs of acute graft-vs-host disease (GVHD), was also applied successfully for the prevention of relapse by pre-emptive DLI in patients with high-risk disease (Naparstek et al. 1995). Unfortunately, whereas DLI treatment following allogeneic SCT for treatment of acute and chronic hematologic malignancies could sometimes eliminate resistant malignant cells, durable circulation of donor lymphocytes following induction of transplantation tolerance by engraftment of donor’s stem cells was always risky due to unpredicted toxicity, morbidity and mortality due to unavoidable acute and chronic GVHD. Moreover, the mandatory immunosuppressive treatment indicated for prevention or treatment of established GVHD following SCT also impaired the efficacy of potential GvL effects. Taken together, unfortunately, despite partially successful attempts to apply DLI for treatment of early relapse following allogeneic SCT (Collins et al. 1997; Kolb et al.^1995^; Mackinnon et al. 1995; Slavin and Nagler 1991; Slavin et al. 1995, 1996; Takahiro et al. 2017), the GvL effects induced by MHC-compatible lymphocytes obtained from fully HLA-matched donors was not sufficiently effective for control of overt and fast progressing relapse, not even when the intensity of GvL resulted in life-threatening acute GVHD.

In an attempt to maximize the anti-cancer capacity of GvL effects that could be induced by MHC-compatible donor lymphocytes we used our spontaneous murine B cell leukemia (BCL1) (Slavin and Strober 1978) first documenting that even the GvL-like effects induced by syngeneic donor lymphocytes could be amplified by activation with interleukin 2 (IL-2) (Slavin et al. 1988; Ackerstein 1991). Subsequently, the opportunity to document similar enhancement of GvL effects by IL-2-activated DLI following super-myeloablative conditioning and allogeneic SCT from a fully matched sibling was confirmed in late 1986 when a patient with fully resistant relapsed ALL following super-myeloablative conditioning was rescued with IL-2 activated DLI after failing to respond to treatment with naïve donor lymphocytes (Slavin and Nagler 1991; Slavin et al. 1995). This patient is alive and well today, more than 36 years later, with no further treatment, luckily with no acute or chronic GVHD. Unfortunately, although IL-2 activated DLI could induce stronger GvL effects in additional patients that failed to responds to DLI induced by naïve donor lymphocytes, activation of DLI was frequently associated with much more risky acute and chronic GVHD.

It seemed obvious that safer and smarter cell-mediated immunotherapy is highly indicated for treatment of patients with MDR hematologic malignancies. Accordingly, we have used several animal models in an attempt to develop safer and more effective approaches for improving the anti-cancer effects inducible by alloreactive donor lymphocytes while avoiding the need for risky SCT and the consequences of acute and chronic GVHD induced by durable engraftment of alloreactive donor lymphocytes.

The feasibility to amplify the therapeutic effects of immunotherapy by the use of haploidentical or fully mismatched donor lymphocytes activated with IL-2 was first confirmed in pre-clinical animal models. Using BALB/c mice inoculated with lethal doses of BCL1 and SJL/J mice inoculated with lethal doses of acute murine myeloid leukemia, we confirmed that treatment with IL-2-activated MHC mismatched spleen cells obtained from C57BL/6 donors resulted in complete eradication of both lymphocytic (Weiss et al. 1992) and myeloid leukemia (Vourka-Karussis et al. 1995). Interestingly, elimination of a lethal challenge of BCL1 inoculated in (BALB/c X C57BL/6)F1 mice following infusion of recipients with IL-2 activated C57BL/6 lymphocytes resulted in cure, in sharp contrast to similar treatment using IL-2 activated BALB/c spleen cells, although both IL-2 activated BALB/c or C57BL/6 spleen cells could result in equal GVHD against F1 recipients (Cohen et al. 1993). These observations indicated that effective anti-cancer effects were induced by alloreactivity against mismatched target cells and not by the non-specific alloreactivity induced by GVHD per se. Taken together, it became obvious that much more effective and much faster GvL-like anti-cancer effects could be induced by transient circulation of IL-2 preactivated intentionally mismatched killer cells consisting of a mixture of both T and NK cells. Similarly, as will be reported separately, the use of short-term circulation of intentionally mismatched killer cells, including both T and NK cells, could also rescued mice inoculated with lethal doses of metastatic breast cancer and metastatic malignant melanoma (Slavin et al., manuscript in preparation).

We hypothesized that due to much more effective and faster induction of anti-cancer effects inducible by mismatched IL-2 activated killer cells, elimination of malignant cells could be accomplished within a few days by non-engrafting donor lymphocytes, thus avoiding the need for prior SCT to ensure consistent rejection of mismatched donor lymphocytes after induction of cytotoxicity against the malignant cells for prevention of GVHD. Accordingly, it seemed reasonable to assume that clinical application of intentionally mismatched activated killer cells (IMAK) at the stage of minimal residual disease (MRD) or against a low tumor burden could similarly result in complete elimination of all multi-drug resistant malignant cells, including cancer stem cells. In 1993 we had the first opportunity to confirm our working hypothesis in a patient with fully resistant AML after failure of conventional chemotherapy and myeloablative autologous SCT (Slavin 2005). This patient is currently alive and well with no further treatment, now nearly 30 years. Since then, we have applied our new IMAK immunotherapy program for the compassionate treatment of otherwise incurable patients fully resistant to all available conventional modalities. The present investigations represent the cumulative experience of compassionate treatment of 33 patients treated with IMAK.

## Patients and methods

### Patients

A total of 33 consenting patients, 21 males and 12 females, aged 3–63 (median 30) with different hematopoietic malignancies detailed in Table 1 were treated with IL-2 activated lymphocytes obtained from haploidentical or fully mismatched unrelated consenting donors on a compassionate basis between 1993 and 2016 due to multi-drug resistant relapse. Treatment protocol was approved by the Ethical Committee of the Hadassah Medical Center & University Hospital in Jerusalem, Israel, and each patient signed an approved informed consent according to the principles expressed in the declaration of Helsinki.

**Table 1:** Diagnosis of patients with advanced multi-drug resistant hematologic malignancies treated with IMAK

Multiple myeloma	2
Non-Hodgkin lymphoma	9
AML	8
ALL	14
Total	33

### Preparation of activated donor lymphocytes

Peripheral blood lymphocytes were obtained by aphaeresis using a COBE spectra cell separator from haploidentical related donors (*n* = 20) or unrelated volunteers (*n* = 13). Cells were transferred into 250 ml tubes and loaded over ficoll gradient at room temperature and spun for 20 min at 2000 rpm using a Sorval centrifuge. Cells were cultured in a 1 L Lifecell bag (Baxter, USA) at a concentration of 2 × 10^6^ cells/ml in RPMI medium supplemented with 10% heat-inactivated human AB serum, glutamine 1%, and antibiotics (Gentamicin 0.1%). Recombinant human IL-2 (Proleukin, The Netherlands) was added at 6000 IU/ml. Cells were placed in a 5% CO_2_ in air incubator at 37 ℃, at a maximum volume of 1500 ml in 3 L Lifecell bags for 4 days. Activated lymphocytes were harvested following 4 days of culturing. Upon termination of incubation, cells were transferred to a 600 ml transfer pack by plasma transfer set. Cells were spun at 1200 rpm for 15 min, washed and resuspended in saline in a 150 ml transfusion bag ready for infusion.

IMAK enriched for NK cells was used in 3 patients with AML. CD56 positive NK cells were selected using Miltenyi’s immunomagnetic bead system (Miltenyi Biotec GmbH, Bergisch Gladbach, Germany). Positively selected NK cells consisted of 30–71% CD56^+^ (median 39%) cells and 2–21% CD3^+^ (median 3%) T cells.

IMAK infusion was combined with infusion of Rituximab 100 mg in an attempt to target Fc receptor-positive NK cells against malignant B cells in 7 patients with ALL and 5 patients with NHL.

### Conditioning of patients before cell infusion

Conditioning was applied by a single intravenous cyclophosphamide 1000 mg/m^2^ on day 1 with forced hydration 3L/m^2^ before infusion of IMAK on day 0. Only 4 patients were pre-treated with heavier immunosuppressive conditioning as previously described (Slavin et al. 2010).

Starting with the conditioning, all patients were treated with prophylactic anti-inflammatory indomethacin 25 mg tablets × 2/day and ranitidine (Zantac) tablets 150-300 mg/day to minimize adverse reactions COX 2-induced fever and inflammatory reactions that could result from infusion of IL-2-activated killer cells followed by low-dose IL-2 administration following cell infusion. Just before cell infusion patients received promethazine 12.5 mg against any potential hypersensitivity reaction.

### Cell therapy based on the use of IMAK

A total of 1.5–18.0 (median 3.0) × 10^7^ cells/kg were slowly injected intravenously on day 0. A total of 7 patients with CD20-positive ALL out of a total of 14, and 5 patients out of 9 with CD20-positive non-Hodgkin lymphoma were treated with IMAK targeted against the malignant cells using anti-CD20 monoclonal antibody (Rituximab) together with cell infusion.

### Treatment of patients after cell infusion

Starting on day 0, subcutaneous injections of low doses IL-2 (< 6 × 10^6^ IU/m^2^) individually adjusted to prevent adverse reactions for up to 5 consecutive days to continue activation of killer cells until anticipated rejection.

### Engraftment of donor lymphocytes and GVHD following treatment with IMAK

All patients were closely monitored for toxicity and clinical and laboratory signs of acute and later on for possible chronic GVHD. Chimerism in female recipients of male cells was checked by cytogenetic analysis and later using male-specific amelogenin gene by PCR as previously described (Pugatsch et al. 1997). Donor-specific VNTR-PCR was used in female- to-female, male-to-male, or female-to-male cases for detection of circulating donor cells to confirm complete rejection of mismatched IMAK.

### Long-term observations of patients

All patients were observed for signs of toxicity, acute and chronic GVHD and possible recurrent disease as long as they were under our observation.

### Data sharing statement

Individual participant data will not be shared but identified individual participant data will be available in the filed medical records available at the Hadassah University Hospital in Jerusalem.

## Results

### Procedure-related toxicity and risk of GVHD

As shown in Table 2, conditioning before cell infusion was accomplished with acceptable toxicity with no grade 4 among the first cohort of 33 heavily pre-treated patients with different resistant hematologic malignancies. Conditioning with cyclophosphamide alone was much simpler and much better tolerated among 29 patients in comparison with a previous cohort of 4 patients pre-treated with heavier immunosuppressive conditioning (Slavin et al. 2010). Also, the only suspected 2 cases of possible grade I GVHD occurred among the patients pre-treated with heavier immunosuppressive conditioning. Administration of IL-2 following cell infusion was accompanied by local erythema at the site of injection in 22 patients, and malaise or mild fever responded to anti-inflammatory agents. Skin erythema compatible with grade I GVHD was observed in 2 patients. A total of 16 patients, 8 with grade II and 2 with grade III developed significant fever and/or chills requiring additional treatment. Other adverse reactions are shown in Table 2 with grade III observed in 14 patients but none with grade IV. As shown in Table 2, patients conditioned with mild cyclophosphamide conditioning had less severe adverse reactions as compared with patients in a previous cohort pre-treated with more immunosuppressive conditioning (Slavin et al. 2010).

**Table 2:** Adverse reactions of all 33 patients treated with IMAK following mild or moderate immunosuppressive conditioning

	Grade 0	Grade I	Grade II	Grade III	Grade IV
Hemoglobin	23	4	6	0	0
Leucocytes	19	6	6	2*	0
Platelets	14	7	9	3*	0
ALT	22	4	5	2*	0
AST	24	4	3	2*	0
GGTP	27	1	2	3*	0
ALK phosphatase	28	3	2	0	0
Erythema at IL-2 injection site	11	20	2^†^	0	0
Fever and/or shaking chills	17	6	8	2*	0
Skin erythema; grade I GVHD?	31	0	2*	0	0

*Adverse reactions among patients conditioned with more aggressive lymphoablative conditioning (16)

^†^Dose reduction of IL-2 injections due to fever and malaise and reduction of treatment to 3 days instead of 5 days subcutaneous IL-2 injections was indicated in 8 patients

All procedures were carried out in the outpatient setting and no patient required admission to the hospital.

Due to subjective intolerance to IL-2, the daily dose was reduced to 0.5 × 10^6^ IU and IL-2 administration was reduced from 5 to 3 days in 8 patients. Importantly, apart from the adverse reactions shown in Table 2, no patient developed any sign of clinically significant acute GVHD and no patient developed any sign of chronic GVHD, due to consistent rejection of donor lymphocytes, since circulating donor lymphocytes were never detected beyond day + 6.

### Anti-cancer effects induced by IMAK

**Fig. 1:**
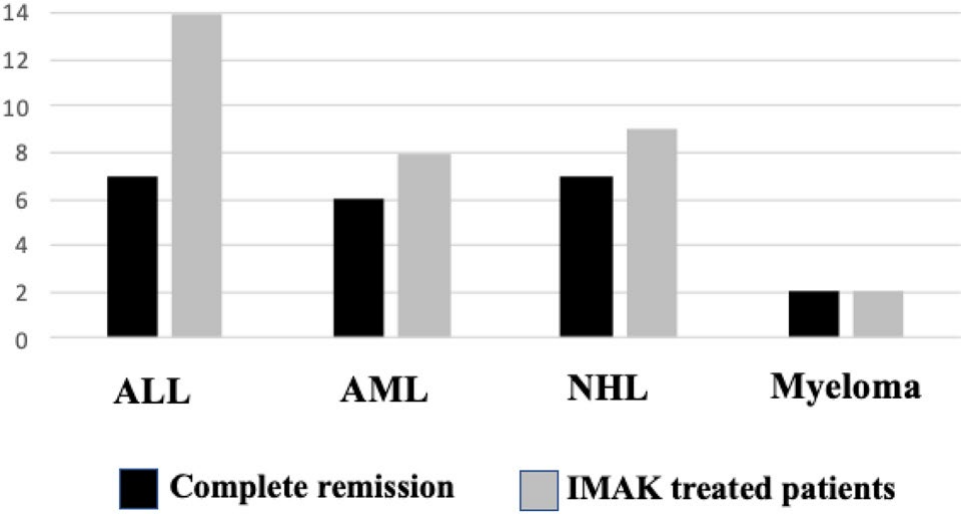
Successful induction of complete remission, progression-free survival or possibly even cure, in patients with different types of multi-drug resistant hematologic malignancies following treatment with intentionally mismatched IL-2 activated killer cells (IMAK)

Details and outcomes following cell therapy with IMAK are shown in Table 3 and summarized in Fig. 1. Interestingly, the anti-cancer effects of IMAK seemed to be equally effective against all hematologic malignant cells investigated including lymphoid, myeloid and malignant plasma cells. Out of the first cohort of 33 patients treated with IMAK for recurrent multi-drug resistant disease a total of 22 were with confirmed complete response, 5 with partial response or very good partial response, and only 6 with progressive disease. The first patient treated with IMAK at the age of 12 years is currently 30 years out, a physician in perfect clinical condition with no further treatment since then happily married with 2 children. Following the diagnosis of acute promyelocytic leukemia, complete remission could not be accomplished in the Philippines and residual disease was confirmed following chemotherapy in a major medical center in Los Angeles. Accordingly, the patient was referred to the Hadassah Medical Center in Jerusalem but residual AML was confirmed even following myeloablative hematopoietic stem cell transplantation. Therefore, it was suggested to try an alternative cell-mediated immunotherapy using intentionally mismatched lymphocytes as supported by the pre-clinical investigations detailed above, first using unsuccessfully DLI infusions using naïve maternal lymphocytes. Next, complete eradication of all detectable AML was only accomplished following treatment with IMAK, using maternal lymphocytes preactivated with IL-2 for 4 days prior to cell infusion and with subcutaneous injections of IL-2 for 3 days following cell infusion. Accordingly, a similar therapeutic strategy was applied as compassionate treatment in the next cohort of 32 patients considered incurable by any available conventional procedure. Out of the 22 responders that can be seen in Table 3, at least 6 observed for > 5 to > 30 years with no further treatment should be considered cured. All 6 patients with progressive disease were treated with IMAK at the time they presented with progressive disease.

**Table 3:** Outcome of all 33 patients with multi-drug resistant hematologic malignancies treated with IMAK for the past 30 years

	Type of cell therapy	Number of patients treated	Number of patients in CR, PFS or cure	Number of patients with PR or VGPR	Number of patients with PD
Relapsed resistant ALL	IL-2 activated mismatched PBL	7	3	1	3
Relapsed resistant ALL	IL-2 activated mismatched PBL + anti-CD20 monoclonal antibody	7	4	2	1
Relapsed resistant AML*	IL-2 activated mismatched PBL	5	3	1	1
Relapsed resistant AML	IL-2 activated mismatched NK cells	3	3		
Relapsed non-Hodgkin’s lymphoma	IL-2 activated mismatched PBL	4	4		
Relapsed non-Hodgkin’s lymphoma	IL-2 activated mismatched PBL + anti-CD20 monoclonal antibody	5	3	1	1
Advanced relapsed multiple myeloma (one after HSCT)	IL-2 activated mismatched PBL	2	2		
Total number of patients treated with IMAK		33	22	5	6

*AML* acute myeloid leukemia, *ALL* acute lymphocytic leukemia, *PBL* peripheral blood lymphocytes, *CR* complete remission, *PFS* progression-free survival, *PR* partial remission, *VGPR* very good partial response, *PD* progressive disease, *HSCT* hematopoietic stem cell transplantation

*Including the first patient with resistant AML that failed conventional chemotherapy and myeloablative autologous hematopoietic stem cell transplantation (HSCT) successfully treated with IMAK currently, 30 years later, alive and well with no further treatment, the first that confirmed the feasibility of a cure potential of fully resistant leukemia using IL-2 activated non-engrafting intentionally mismatched donor lymphocytes with no need for prior allogeneic stem cell transplantation and no risk of acute or chronic GVHD

Since only 7 out of 14 patients with ALL and 5 out of 9 with NHL were treated with IMAK targeted against malignant B cells with Rituximab, no information is available if targeting IMAK against B cells with anti-CD20 monoclonal antibodies was contributory. Likewise, since only 3 out of 8 patients with AML were treated with T cell-depleted NK cells-enriched IMAK and none of the 8 developed significant GVHD, no conclusions can be drawn if depletion of T cells is essential to prevent GVHD or if the only effective killer cells are NK cells.

## Discussion

The main purpose of the present investigations was to provide proof of principle of feasibility, safety, and efficacy of a simple and inexpensive protocol for more effective cell-mediated immunotherapy of patients fully resistant to all available anti-cancer modalities. Based on successful cure with no severe side effects of the first 12 years old girl treated with IMAK currently nearly 30 years out with no further treatment, a physician with two children in excellent clinical condition (Slavin 2005), a total of 32 additional patients with different MDR incurable hematologic malignancies not eligible for allogeneic SCT consented to be treated with IMAK. Considering the anticipated short circulation time of non-engrafting mismatched donor-derived killer cells, it seemed reasonable to anticipate that cure could be anticipated only when IMAK will be applied against MRD or against a relatively low tumor burden. Alternatively, the procedure would have to be repeated using different donor cells, but in this pilot study only one treatment cycle with IMAK was applied. Indeed, as shown in Table 3 and Fig. 1, it was really surprising that 22 out of 33 patients treated with IMAK using either haploidentical related donor cells or fully mismatched lymphocytes obtained from consenting unrelated donors showed evidence of response, while at least 6 of 22 patients that were observed for more than 5 years may even be considered cured.

The anti-cancer effects of IL-2-activated lymphocytes is not new but originally considered clinically relevant for activating patient’s own anti-cancer effector cells in patients with cancer (Mule et al. 1984; Rosenberg et al. 1985; Rosenberg et al. 1987). Focusing on the particular role of NK cells as anti-cancer effector cells was also confirmed by many previous investigators (Kärre 2002; Myers and Miller 2021; Chu et al. 2022) and the use of NK cells derived from different possible sources was also suggested by Miller and colleagues (Johnson and Miller 2018; Tanaka and Miller 2020). The important contribution of the present work using IMAK for treatment of multi-drug resistant cancer initially pioneered by Slavin in 1993 for successful treatment of patients with hematologic malignancies (Slavin 2005) and subsequently also for treatment of multi-drug resistant metastatic solid tumors (Slavin et al. 2010) is documentation of the potential safe use of the most effective killer cells for successful treatment and even potential cure of patients considered otherwise incurable. The other special feature of IMAK in comparison with other suggested immunotherapy procedures focusing on the use of isolated NK cells is the easier and less expensive immunotherapy procedure that can be easily applied on a larger scale based on the use of the activation of unseparated lymphocytes collected by apheresis, including a mixture of both fully mismatched T and NK cells preactivated with IL-2 for a very short period of 4 days. Infusion of IMAK was followed by continuous in vivo activation of donor lymphocytes with low-dose subcutaneous IL-2 injections for no longer than 5 days. As such, both donor’s and patient's own NK cells and T lymphocytes can play a role in induction of anti-cancer cytotoxicity and activation of immune-mediated rejection of MHC-incompatible cancer cells, respectively. Induction of forceful immune reaction against MHC-incompatible target cells represents a most effective and consistent biologic principle that was extrapolated by IMAK for maximizing direct anti-cancer cytotoxicity of the one hand, while ensuring consistent rejection of killer cells after induction of anti-cancer cytotoxicity for prevention of GVHD, on the other. The concept of combined use of both T cells and NK cells for cancer immunotherapy was recently upgraded (Slavin, in preparation) toward developing an even more sophisticated immunotherapy procedure based on the combination of elimination of existing cancer cells followed by induction of long-lasting anti-cancer immunity (Morecki et al. 2006, 2008), aiming for induction of resistance against recurrent disease (Slavin, in preparation).

Although our cumulative experience in a small number of patients in need confirmed the feasibility, safety and efficacy of immunotherapy induced by IMAK, many questions remain to be answered. First, the role of IMAK needs to be confirmed in prospective randomized clinical trials for treatment of different disease categories, both for treatment of patients with overt relapse and later on, provided that safety of IMAK treatment will be confirmed, also for patients with high-risk disease with MRD following successful response to conventional treatment. Fortunately, induction of complete remission can be accomplished by conventional 1st line chemotherapy even in patients with high-risk leukemia or lymphoma at an early stage of the disease, or if indicated following high-dose chemotherapy and supportive autologous SCT, as per the routine procedure in patients with multiple myeloma. Therefore, once the safety and efficacy of IMAK will be confirmed, eradication of MRD potentially resulting in cure could be easily accomplished at an early stage of high-risk disease before relapse occurs, when another opportunity to accomplish MRD against mutated and more resistant malignant cells may be difficult or impossible to be accomplished.

Other important parameters that need to be investigated include whether treatment outcome could be improved by targeting the mismatched killer cells with relevant monoclonal antibodies against cancer-associated cell surface antigens such as CD20, CD19, CD38 to mention just a few. Also, as shown in Table 3, using NK-enriched mismatched killer cells for treatment of 3 patients with AML was also successful, suggesting that IL-2-activated NK cells may be the dominant anti-cancer effector cells. Using CD3-depletion or NK-enriched CD56 positive killer cells will certainly minimize any potential risks of GVHD in patients that may remain immunosuppressed by prior anti-cancer modalities or SCT, since an extended circulation time of IMAK is likely to increase the risk of GVHD. Accordingly, we need to investigate if treatment with T cell-depleted or NK-enriched killer cells is worth the extra-complicated procedure and higher cost as compared with using an unmanipulated mixture of IL-2 activated killer cells.

Another potentially important question is whether the anti-cancer effects inducible by IMAK could be improved by control of patient’s negative regulators such as regulatory T cells, checkpoint inhibitors, and myeloid-derived suppressor cells. Also, in the special case of diseases caused by malignant B cells or multiple myeloma, the anti-cancer effects induced by IMAK will have to be compared to the anti-cancer effects induced by CAR-T cells. More important, it would be interesting to investigate if recurrent disease following treatment with CAR-T cells can be controlled by IMAK.

Taken together, the concept of fast and effective killing of malignant cells despite resistance to maximally tolerated doses of chemo-radiotherapy and other available anti-cancer modalities by cell therapy alone, while avoiding much more complicated allogeneic SCT and the risks of GVHD, could possibly represent a new approach for more effective yet much simpler approach for immunotherapy of otherwise incurable hematologic malignancies. Our available proof of principle should provide the basis and justification for future investigations and prospective clinical trials to confirm the safety and challenge the efficacy of cell-mediated immunotherapy based on the use of intentionally mismatched donor lymphocytes.

Remembering that no available immune reaction can compete with the capacity of mismatched lymphocytes to reject any MHC mismatched target cells, including even kilograms of organ allografts in sub-optimally immunosuppressed recipients, our prediction based on multiple studies in pre-clinical animal models and our limited clinical experience suggests that IMAK application against a true stage of MRD could result in complete eradication of all residual resistant malignant cells. Future studies are indicated to prove or disprove our working hypothesis.

## Data Availability

The data analyzed during the current investigations should be available in patient’s files at the Hadassah Hospital in Jerusalem.

## Abbreviations

IMAK: Intentionally mismatched activate killer cells
MDR: Multi-drug resistant cancer
SCT: Stem cell transplantation
DLI: Donor lymphocyte infusions
GVHD: Graft-vs-host disease
GvL: Graft-versus-leukemia
IL-2: Interleukin 2
ALL: Acute lymphoblastic leukemia
AML: Acute myeloid leukemia
NHL: Non-Hodgkin lymphoma
NK: Natural killer cells
CR: Complete remission
PFS: Progression-free survival
